# Multifunctional High-Concentration Polyepoxysuccinic Acid for Water-Based Drilling Fluids: Achieving Ultra-Low Friction and Filtration

**DOI:** 10.3390/polym17060751

**Published:** 2025-03-12

**Authors:** Fuchang You, Yu Wu, Xingguang Gong, Yancheng Zheng

**Affiliations:** 1 College of Chemistry & Environmental Engineering, Yangtze University, Jingzhou 434023, China; yfc81@yangtzeu.edu.cn (F.Y.);; 2 School of Petroleum Engineering, National Engineering Research Center for Oil & Gas Drilling and Completion Technology, Yangtze University, Wuhan 430100, China; 3 Mud Service Company of CNPC Bohai Drilling Engineering Co., Ltd., Tianjin 300280, China; cnpcgxg@126.com

**Keywords:** water-based drilling fluid, polyepoxysuccinic acid, friction, filtration loss

## Abstract

Water-based drilling fluids (WBDFs) cannot be effectively applied in long horizontal wells, such as shale gas wells, due to their high coefficient of friction (COF) and filtration loss that can strongly limit the efficient and environmentally friendly development of oil and gas resources. The objective of this study is the formulation of a WBDF characterized by ultra-low friction and ultra-low filtration properties, with a high-concentration polyepoxysuccinic acid (PESA) solution being utilized in the continuous phase. The research aims at the exploration of the feasibility of the method, the validation of the results, and the elucidation of the underlying mechanisms. The experimental results confirmed that the proposed WBDFs have excellent rheological properties, a COF of 0.016 and an API filtration of 0.4 mL. Microscopic analysis confirmed a direct and positive correlation between the macroscopic properties of the drilling fluids and their adsorption behavior at high PESA concentrations. This approach can be used to redesign traditional WBDFs and provide new possibilities to realize super performance in WBDFs that can be used to replace oil-based drilling fluids.

## 1. Introduction

Conventional oil and gas resources are easily exploited, but their outputs show a declining trend; therefore, they will not be able to meet the increasing demand for oil and gas over the next few decades [1]. Shale gas has gradually become an important supplement to global oil and gas resources with its rapid development as an unconventional oil and gas resource in the United States [2,3]. To improve efficiency and save costs, the number of long-reach wells and long horizontal wells applied in conventional oil and gas production is gradually increasing, and long horizontal wells are often used in unconventional oil and gas production for efficient development, which could pose substantial technical challenges for drilling fluids [4,5]. Drilling fluids are categorized into three types: water-based drilling fluids (WBDFs), oil-based drilling fluids (OBDFs), and synthetic-based drilling fluids (SBDFs) [6,7]. OBDFs and SBDFs are the preferred for the development of long-reach wells and long horizontal wells due to their low coefficients of friction (COF) and minimal filtration losses. However, the risk of OBDF or SBDF leakage and the subsequent treatment of drill cuttings pose threats to the environment, which restricts their widespread application of OBDFs and SBDFs [8,9]. Compared with OBDFs or SBDFs, WBDFs have been widely used for drilling operations and have good environmental safety; however, their lubricity and filtration control performance are inferior to OBDFs or SBDFs, and WBDFs cannot be used to resolve field problems such as wellbore instability and excessive torque [10,11,12]. For more than ten years, in the large-scale development of shale gas in China, researchers have been working to develop WBDFs with ultra-low COF and filtration losses, to replace OBDFs or SBDFs. Although several hundred wells have been tested, the results were poor and have not been publicly reported.

WBDFs consist of complex suspension and dispersion systems containing water, polymers, water-soluble salts, and water-dispersed solids, and their macro properties, such as their rheology, filtration, and lubricity, can be altered by the addition of various materials such as cellulose and polyacrylamide [13,14]. To achieve rheological properties that are suitable for drilling hydraulic conditions [15], researchers typically synthesize or optimize various chemical materials to improve the lubricity and filtration control performance of WBDFs [16,17]. When the material concentrations are generally less than 5%, the macro performance of WBDFs is effectively improved and stabilized with increasing material concentration [18,19]. On the other hand, a further increase in material concentration deteriorated the other properties of these fluids, such as rheological properties [20]. Therefore, low concentrations of materials are added to drilling fluid systems. High concentrations of water-soluble salt or water-insoluble salt materials, such as sodium chloride, sodium formate, barium sulfate, and micro-manganese powder, are used solely to increase the density of drilling fluids [21,22]. Traditional drilling fluid design approaches significantly constrain the possibility of achieving high performance in WBDFs, and new approaches must be developed and applied to prepare WBDF systems.

High concentrations of materials are utilized to achieve high-performance in various industries [23,24,25]. In this study, the objective was to explore whether a similar approach could be used to improve the performance of WBDFs such that they performed better than OBDFs and SBDFs. Common small molecules such as inorganic or organic salts can increase the density of the fluids only at high concentrations, and high-molecular polymers have high viscosity at low concentrations. Therefore, neither small molecules nor high-molecular polymers can be used to achieve excellent performance in WBDFs. A water-soluble polymer with an appropriate molecular weight can be used to achieve excellent performance, characterized by an ultra-low COF and reduced filtration loss in WBDFs. The polyepoxysuccinic acid (PESA) used in the mineral flotation and water treatment industry exhibit strong adsorption and are environmentally friendly [26,27,28,29], increasing the feasibility of realizing excellent performance in water-based drilling fluids that can replace oil-based drilling fluids.

To achieve this goal, this is the first report of a new approach for designing super-performance WBDFs (SP-WBDFs) based on a high concentration of PESA, presenting a new model for the future design of WBDFs systems with super performance and promoting the advancement of materials and technologies for WBDFs. Therefore, the objectives of this study are as follows: (1) Explore the feasibility of this method, (2) form a SP-WBDF based on high-concentration PESA, (3) determine the relationship between the friction performance of WBDFs and the concentration of PESA, and (4) elucidate the possible mechanism of high-concentration PESA in WBDFs.

## 2. Materials and Method

**Figure 1:**
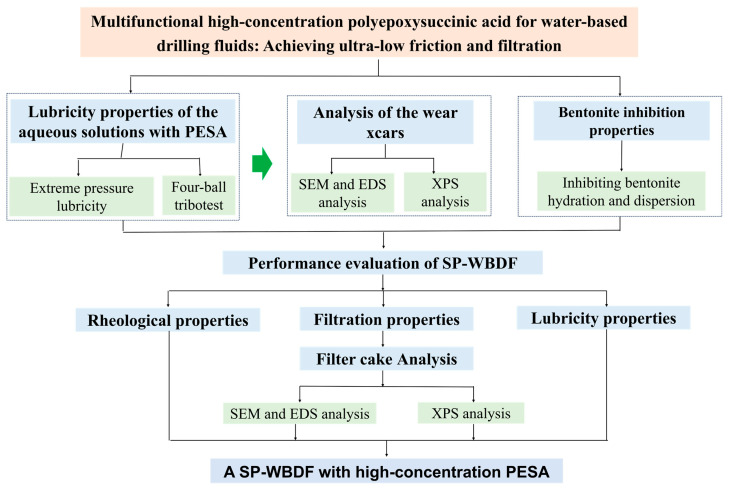
Flow chart of the experimental procedure.

This paper reveals the mechanism of ultra-low friction and filtration loss with PESA, and the super-performance WBDFs (SP-WBDFs) with high-concentration PESA were systematically studied. The following flow chart shows the experimental process for the objective of this study, as shown in Figure 1.

### 2.1. Materials

As a key additive for drilling fluids, polyepoxysuccinic acid (PESA), which has a molecular weight between 400 and 1500 g/mol, was obtained from Shandong Taihe Technology Co., Ltd., Zaozhuang, China. and was used to alter the lubricity and filtration properties of drilling fluids. Chemically pure sodium hydroxide (NaOH) was used to control the pH of the drilling fluid and was obtained from Sinopharm Chemical Reagent Co., Ltd., Shanghai, China. Polyanionic cellulose (code PAC-LV) was purchased from Jiangsu Telida New Materials Co., Ltd., Nantong, China. Xanthan gum was purchased from Ordos Zhongxuan Biochemical Co., Ltd., Ordos, China. Pregelatinized corn starch (code starch) was obtained from Shandong Rongda Starch Co., Ltd., Weifang, China. Partially hydrolyzed polyacrylamide (code PHPA) was supplied by Shandong Nuer Biological Technology Co., Ltd., Dongying, China, and its degree of hydrolysis and average molecular weight were 27.4% and 1.0 × 10^7^ g/mol, respectively. Sodium bentonite was purchased from Shandong Weifang Damei Bentonite Co., Ltd., Weifang, China. Barite with a density of 4.2 g/cm^3^ was purchased from Anxian Huaxi Mineral Powder Co., Ltd., Mianyang, China, and mineral oil was obtained from Zhonghai Nanlian Co., Ltd., Maoming, China.

### 2.2. Extreme Pressure Lubricity Test

According to international standards, API RP 13B-1 (2009) [30] and ISO 10414-1 (2008) [31], the COF of aqueous solutions containing different concentrations of PESA as an additive was measured via an extreme pressure lubricity tester (Model 212, Fann Instrument Company, Houston, TX, USA). The lubrication ring and slider, as the friction-inducing parts of the instrument, were designed to simulate a drill string and a borehole. During the dynamic friction test, the slider was pressed against the rotating ring by an external force. A total torque of 150 inches was applied to the slider, and the rotation speed of the ring was kept constant at 60 rpm. The torque was read after 5 min. The experiment was repeated 3 times at 25 °C. The COF of the aqueous solutions can be calculated to characterize the lubricity of PESA, as shown in Equation (1).

(1) $$k_{0}=\frac{34}{k_{w}}k_{d}/100$$

where k_w_ and *k*_d_ are the measured torque readings in deionized water and the PESA-containing aqueous solution, respectively, and *k*_0_ is the COF of the PESA-containing aqueous solution.

### 2.3. Four-Ball Tribotest

The four-ball friction test method, referenced from the literature and appropriately modified [32], was performed using a four-ball testing machine (Model SGW-10 W, Jinan Hengxu Testing Machine Technology Co., Ltd., Jinan, China) to evaluate the tribological performance of aqueous solutions containing different concentrations of PESA. The machine consisted of a shaft at the top and a ball holder at the bottom. The shaft held and rotated the top ball, and the ball holder fixed three stationary balls. The diameter of the four balls was 12.7 mm. The aqueous solution was dropped into the contact zone of the balls. The test was conducted by rotating the top ball at a constant speed under a constant test load on the three stationary balls (load: 400 N; rotation rate: 120 rpm; time: 30 min; temperature: 25 °C). The changes in the COF of the aqueous solutions as a function of the test duration were automatically recorded by the machine.

### 2.4. Bentonite Inhibition Test

The bentonite inhibition test with the aqueous solutions containing various concentrations of PESA was based on the general procedure described by Estupiñán [33]. At room temperature, sodium bentonite was added to 1000 mL of the aqueous solutions containing PESA, and the amounts of sodium bentonite used were 100, 200, 300, 400, 500, and 600 g. The mixtures were stirred at 10,000 rpm for 20 min.

### 2.5. Preparation of the SP-WBDFs

The WBDFs were prepared in accordance with the national standard (GB/T 16783.1-2014) [34]. The following steps were used to prepare it: fresh water and PESA were first added to a mud cup and stirred for 10 min to produce a uniform aqueous solution as the base fluid for the drilling fluids; then, NaOH particles were added to the cup to adjust the pH value of the base fluid and stirred for 5 min; next, xanthan gum, PAC-LV, starch and PHPA, the main components of the prepared drilling fluid, were added to the above cup at 10 min intervals with stirring; and finally, barite was added to the mixture, followed by stirring for 30 min to produce the drilling fluid. During preparation, the fluid was stirred at a speed of 10,000 rpm at ambient temperature. The prepared drilling fluid was placed in a roller heating furnace (XGRL-4, Qingdao Haitongda Special Instrument Co., Ltd., Qingdao, China), aged at 100 °C for 16 h, and then taken out and cooled to ambient temperature. The proportions of the above additives in the fluid are shown in Table 1. Both an OBDF and a SBDF were obtained from shale gas drilling sites in Sichuan, China.

### 2.6. Rheological Measurement

According to the international standards API RP 13B-1 (2009) [30] and ISO 10414-1 (2008) [31], the rheological parameters, including apparent viscosity (AV), plastic viscosity (PV), yield point (YP), and static gel strength (GEL_10m_ and GEL_10s_) of the aged drilling fluid, were measured with a six-speed rotational viscometer (Fann35, Fann Instrument Company, Houston, TX, USA) at ambient temperature. Similarly, the AV, PV, and YP of the PESA solutions with bentonite were measured to assess bentonite inhibition properties in accordance with the aforementioned standards. The calculation formulas are shown in Equations (2)–(6). The rheological properties were measured three times and averaged to ensure consistency.

AV = θ_600_/2 (mPa·s) (2)

PV = θ_600_ − θ_300_ (mPa·s) (3)

YP = 0.511 × (θ_300_ − PV) (Pa) (4)

GEL_10m_ = 0.511 × θ_3m_ (Pa) (5)

GEL_10s_ = 0.511 × θ_3s_ (Pa) (6)

θ_600_ is the dial readings at 600 r/min.

θ_300_ is the dial readings at 600 r/min.

θ_3m_ is the maximum dial reading at 3 r/min with 10 min rest.

θ_3s_ is the maximum dial reading at 3 r/min with 10 s rest.

### 2.7. Filtration Measurement

According to the international standards API RP 13B-1 (2009) [30] and ISO 10414-1 (2008) [31], an API filtration test of the drilling fluids after aging was performed with a medium-pressure filtration apparatus (Model ZNS-3, Taifeng Petroleum Instrument Company, Qingdao, China). The API filtration test was carried out for 30 min at room temperature, and the pressure difference between the inside and outside of the filter cartridge was 0.7 MPa. A high-temperature and high-pressure filtration (HTHP filtration) test of the drilling fluids after aging was performed with an HTHP static filtration apparatus (Model GGS71-B, Qingdao Haitongda Special Instrument Co., Ltd., Qingdao, China). The HTHP filtration test was carried out for 30 min at 100 °C, and the pressure difference between the inside and outside of the filter cartridge was 3.5 MPa. Each experiment was repeated three times under the same conditions to ensure consistency.

### 2.8. SEM and EDS Analysis

After the four-ball tribotest, the wear scars on the balls were first cleaned with heptane and ethanol and subsequently dried in hot air. Next, SEM and EDS were carried out to investigate the scar diameter and morphology and the elemental composition of the wear spot surfaces via a field-emission scanning electron microscope equipped with an energy dispersive spectrometer (Model MIRA-LMS, Tescan Company, Brno, Czech Republic). In addition, the microscopic morphology and elemental composition of the dried filter cakes were analyzed by SEM. An acceleration voltage of 15 keV was used for the SEM-EDS analysis.

### 2.9. XPS Analysis

XPS was performed on the surfaces of the wear spots and the dried filter cakes via an X-ray photoelectron spectroscope (Model Thermo Scientific K-Alpha, Thermo Fisher Scientific Company, Waltham, MA, USA) to investigate their elemental compositions. XPS analysis was performed at pressures less than 2.0 × 10^−7^ mbar, and the X-ray source was Al Kα (hv = 1486.6 eV). Survey spectra were taken with a pass energy of 150 eV and an energy step of 1 eV to identify the elements present on the surfaces of the wear spots and the dried filter cakes. High-resolution spectra were obtained with a pass energy of 50 eV and an energy step of 0.1 eV to more precisely analyze the chemical state of each element.

## 3. Results and Discussion

### 3.1. Lubricity Properties of the Aqueous Solutions with PESA

**Figure 2:**
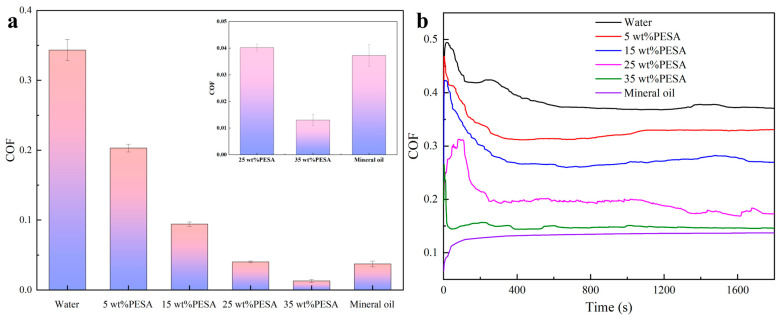
COFs of the fluids with different concentrations of PESA compared with that of mineral oil: (**a**) COF values measured at 150 pounds after 5 min, and (**b**) COF curves recorded in a four-ball tribotest.

To verify the super performance at high concentrations of the material, two experimental methods were employed to evaluate the lubricity properties of the aqueous solutions containing varying concentrations of PESA, with deionized water and mineral oil (as a base oil for OBDFs) as reference samples. The two experimental methods were conducted using an extreme pressure lubricity tester widely used in the drilling fluid industry and a four-ball testing machine commonly applied in the lubricant industry. Figure 2 shows the COFs of the aqueous solutions with different concentrations of PESA. The COFs tested by the extreme pressure lubricity tester are displayed in Figure 2a, while the COF curves tested by the four-ball testing machine are displayed in Figure 2b. The COFs of the aqueous solutions showed a clear decreasing trend as the PESA concentration increased. As shown in Figure 2a, the COF of the aqueous solution with 25 wt% PESA was similar to that of the mineral oil reference sample, while the COF of the aqueous solution with 35 wt% PESA was 0.013, which was much lower than the value of 0.037 for mineral oil. As shown in Figure 2b, the COF of the 35 wt% PESA aqueous solution was slightly greater than that of the mineral oil, which was different from the results of the extreme pressure lubricity test. These differences in results may be caused by differences in the test methods used. However, the overall conclusion was that the aqueous solution with 35 wt% PESA exhibited excellent lubricity.

### 3.2. Analysis of the Wear Scars

**Figure 3:**
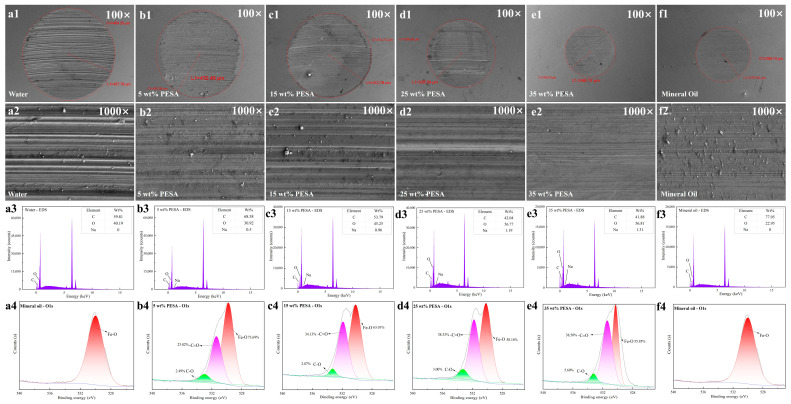
SEM, EDS, and XPS analysis of the wear scars on balls: (**a1**–**a4**) water, (**b1**–**b4**) 5 wt% PESA solution, (**c1**–**c4**) 15 wt% PESA solution, (**d1**–**d4**) 25 wt% PESA solution, (**e1**–**e4**) 35 wt% PESA solution, (**f1**–**f4**) mineral oil.

To explore the lubrication mechanism of the aqueous solutions with high PESA concentrations, SEM and EDS were carried out to analyze the scar diameter and morphology and the elemental composition of the wear spot surfaces. The SEM images and EDS spectra of the wear scars on the balls are shown in Figure 3, respectively. The scar diameter of the wear spot surfaces decreased significantly with increasing PESA concentration. Interestingly, the lubricity performance of the 35 wt% PESA aqueous solution was better than that of the mineral oil in terms of the scar diameter. Overall, when water was used as a lubricant, the worn surface contained more wear debris and was characterized by the largest wear scar with wide and deep furrows. With increasing PESA concentration, both the size and furrow width of the wear scars decreased, and the amount of wear debris also sharply decreased. Although the size of the wear scar after lubrication with mineral oil as a reference sample was very small, the furrow and the attached debris on the worn surface were more pronounced (Figure 3a1–f1 and Figure 3a2–f2). Based on the above results, a high PESA concentration not only results in an ultra-low COF but also provides excellent anti-wear performance. As shown in Figure 3a3–f3, Na was absent on the worn surface after lubrication with water or mineral oil, while Na was detected on the worn surfaces after lubrication with the aqueous solutions containing different concentrations of PESA, which is consistent with the presence of Na in PESA molecules. Compared to the worn surfaces after lubrication with mineral oil, more corrosion products appeared on the worn surfaces after lubrication with water, with an increase in the O content. Additionally, the contents of both Na and O on the worn surfaces showed increasing trends with increasing PESA concentration, indicating that the amount of PESA adsorbed on the metal surface increased with increasing PESA concentration, which further suggested that the COF of the aqueous solutions could be effectively reduced at high PESA concentrations.

To verify the adsorption behavior of PESA molecules on worn surfaces, XPS was carried out to investigate the elemental composition of the worn surfaces. The O1s XPS spectra of the wear scars are shown Figure 3a4–f4. The curve-fitted XPS spectra of the worn surfaces lubricated with water and mineral oil exhibited a strong and distinctive peak near 530.0 eV, which could be assigned to iron oxides, either Fe_2_O_3_ or Fe_3_O_4_ [35]. The curve-fitted XPS spectra of the worn surfaces lubricated with the aqueous solutions with different concentrations of PESA exhibited three distinctive peaks near 530.0, 531.6, and 533.2 eV, which were attributed to iron oxides, –C=O bonds, and C–O bonds [36], respectively. Due to the presence of PESA, there were obvious peaks attributable to –C=O and C–O in the XPS spectra, indicating the adsorption of PESA molecules on the worn surfaces. Interestingly, with increasing PESA concentration, the areas of the –C=O and C–O peaks gradually increased, while the areas of the Fe–O peak showed the opposite trend, indicating that there were more PESA molecules attached to the metal surface with increasing PESA concentration, thus improving the lubrication and anti-wear performance of the fluid and reducing the formation of iron oxides. In the extreme pressure lubricity test and four-ball tribotest, boundary friction mainly occurred when the tribological behaviors of water, oil, and the PESA solutions were measured with steel–steel contacts [37]. The adsorption capacity for water and alkane molecules on the steel surface was poor [38,39]. In contrast, PESA molecules containing many hydroxyl and carboxyl groups were strongly adsorbed on the steel surface [40], and their long molecular chain structure offered multiple adsorption sites to effectively cover the steel surface. The adsorption film thickness increased with increasing PESA concentration. A high PESA concentration resulted in a certain isolation effect on the friction and wear between the steel surfaces [36], thus achieving an ultra-low COF.

### 3.3. Inhibition Properties of the Aqueous Solutions with PESA

**Figure 4:**
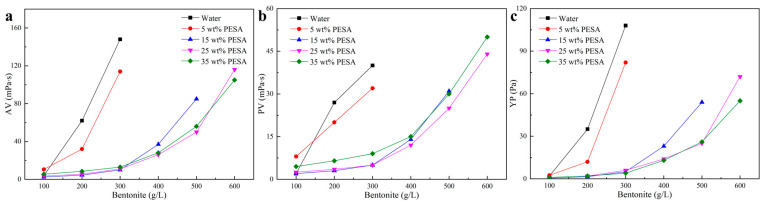
Bentonite inhibition properties of the aqueous solutions with different concentrations of PESA: (**a**) AV, (**b**) PV, and (**c**) YP.

The effects of the aqueous solutions containing different concentrations of PESA on the hydration and dispersion of bentonite were evaluated and compared with those of deionized water. Figure 4 shows the AV, PV, and YP of the PESA solutions contaminated with bentonite. The ability to inhibit the hydration and dispersion of bentonite increased with increasing PESA concentration. Compared with deionized water, the aqueous solution with 5 wt% PESA had a certain ability to inhibit the hydration and dispersion of bentonite, but this ability was not significant. When the amount of PESA was 15 wt%, the ability to inhibit the hydration and dispersion of bentonite became particularly significant. When the amount of PESA exceeded 25 wt%, the AV, PV, and YP of the PESA solutions contaminated with 600 g/L bentonite could still be measured and expressed in the data, which revealed the excellent ability of PESA to inhibit the hydration and dispersion of bentonite. The viscosity and YP of the suspension are related to the number of bentonite particles, their interactions, and the spatial structure formed by the hydration and dispersion of bentonite. Generally, with the increasing number of bentonite particles, the interaction between particles becomes stronger, resulting in an increase in the internal friction [34], which is macroscopically manifested as an increase in the viscosity. There are two possible reasons to explain why the high-concentration PESA can result in a better inhibition performance. On the one hand, high-concentration PESA contains a large amount of sodium ions that can inhibit the hydration of bentonite [41]; on the other hand, due to hydrogen bonds formed by hydroxyl and carboxyl groups, the surface of the bentonite particles can be wrapped by PESA polymer molecules, weakening the hydration effect on both the surface and inside the bentonite particles [42,43]. Cations and polymer molecules can work together to inhibit the hydration and dispersion of bentonite at high concentrations of PESA [44]. The ability of high-concentration PESA to inhibit the hydration and dispersion of bentonite can provide the basis for maintaining stable rheological properties of drilling fluid during well drilling operations.

### 3.4. Performance Evaluation of SP-WBDFs

**Figure 5:**
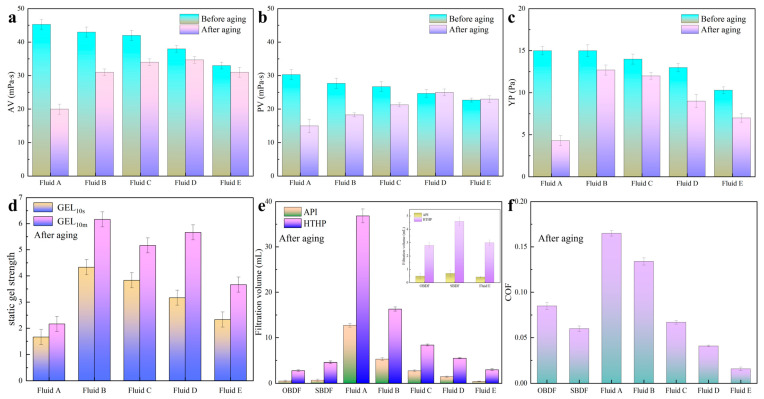
The properties of WBDFs (Fluid A: 0% PESA, Fluid B: 5% PESA, Fluid C: 15% PESA, Fluid D: 25% PESA, Fluid E: 35% PESA): (**a**) AV, (**b**) PV, (**c**) YP, (**d**) static gel strength, (**e**) filtration, and (**f**) COF.

PESA is a well-known green material and has been extensively employed [26,28], and the PESA solutions were used to construct WBDFs in combination with environmentally friendly materials such as PAC-LV, PHPA, pregelatinized corn starch, xanthan gum, and barite [45,46,47]. The formulations of the fluids are listed in Table 1, where Fluid A represents a traditional water-based drilling fluid and Fluids B to E represent WBDFs with different concentrations of PESA. Additionally, both an OBDF and a SBDF were obtained from shale gas drilling sites. All the properties of the drilling fluids were measured, and some of the experimental data are shown in Figure 5. As shown in Figure 5a,b, the AV and PV of the drilling fluids after aging initially increased with increasing PESA concentration and then decreased when the PESA concentration exceeded 25 wt%. As shown in Figure 5c,d, the YP and static gel strength of the drilling fluids after aging initially increased with increasing PESA concentration and then decreased when the PESA concentration exceeded 5 wt%. Compared with those of the drilling fluid without PESA, the rheological parameters of the drilling fluids with different concentrations of PESA changed less before and after aging, indicating that PESA molecules can improve the temperature resistance of polymers such as xanthan gum. The dispersion, flotation, and scale inhibition effects of PESA were due to the adsorption of PESA on the surface of solid particles, which improved the surface properties of the particles and reduced their surface energy so that the solid particles could be more evenly dispersed in the continuous phase [48,49]. Specifically, PESA adsorbed on solid particles, which made the dispersion of solid particles more uniform. Macroscopically, the rheological properties of the water-based drilling fluid with PESA were more stable. The AV, PV, and YP of the water-based drilling fluids slightly decreased at the high PESA concentrations, possibly because the adsorption of PESA at high concentrations reduced the internal friction between solid particles [50]. As shown in Figure 5e, the API filtration and HTHP filtration of the drilling fluids clearly decreased with increasing PESA concentration. When the concentration of PESA reached 35 wt%, the drilling fluid (Fluid E) demonstrated superior filtration performance compared to traditional high-performance WBDFs used for shale gas development. Specifically, the API filtration and HTHP filtration were measured at 0.4 mL and 3.0 mL, respectively, outperforming not only conventional WBDFs, OBDFs, and SBDFs but also previously reported high-performance WBDFs designed for shale gas wells [20,51]. Figure 5f shows the COFs of the drilling fluids. The COF of the drilling fluid with 35 wt% PESA was 0.016, which was lower than the COFs of 0.085 and 0.07 for the OBDF and SBDF, respectively. The extreme pressure COF of the aqueous solution containing 35 wt% PESA was significantly lower than that of mineral oil. Moreover, the extreme pressure COF of the drilling fluid with 35 wt% PESA was markedly lower than those of traditional high-performance WBDFs, OBDFs, and SBDFs reported for shale gas development [52,53,54,55]. With the addition of 35 wt% PESA, both the aqueous solution and the WBDFs showed an ultra-low COF (shown in Figure 2 and Figure 5f), indicating that the lubrication properties at high PESA concentrations were less affected by the drilling fluid additives such as barite particles. These results were obtained mainly due to the strong adsorption of PESA on the surface of solid particles [26,27,28,29]. Under the adsorption of high-concentration PESA, the surface of barite particles and steel may be completely covered by PESA molecules, causing the friction to mainly occur between the adsorbed polymer films, and the wear effect of barite particles on the friction surface was weakened, which was conducive to reducing the COF [56,57,58].

### 3.5. Analysis of the Filter Cake of SP-WBDFs

**Figure 6:**
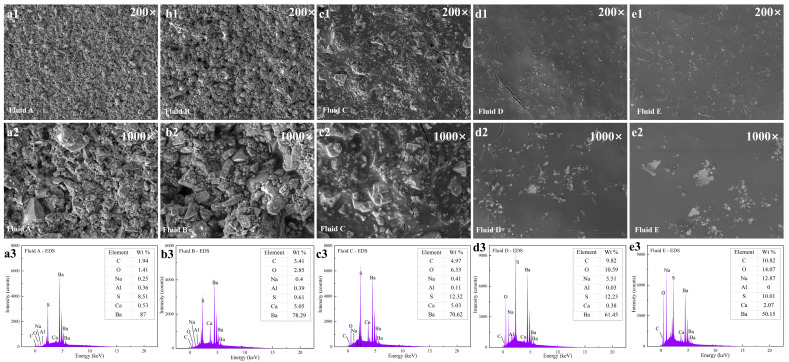
SEM and EDS analysis of the filter cake. (**a1**–**a3**) Fluid A (0% PESA), (**b1**–**b3**) Fluid B (5% PESA), (**c1**–**c3**) Fluid C (15% PESA), (**d1**–**d3**) Fluid D (25% PESA), (**e1**–**e3**) Fluid E (35% PESA).

To study the mechanism by which PESA reduces filtration loss, the microscopic morphology and elemental composition of the filter cakes obtained from the filtration of the five drilling fluids were analyzed by SEM. Figure 6 shows the SEM images and EDS spectra of the filter cakes, respectively. For Fluid A, the accumulation of a solid phase consisting of barite and modified starch particles during filtration was clearly visible, and these solid particles overlapped with each other, resulting in many small pores and a small number of large pores in the filter cake, which was not conducive to controlling the filtration loss (Figure 6a1,a2). With increasing PESA concentration, the pores between the solid phases were filled with PESA, and the pores gradually disappeared on the surface of the filter cakes. When the PESA concentration was greater than 15 wt%, no pores appeared on the surface of the filter cakes, which was the result of the combined action of the PESA molecules and other solid particles (Figure 6b1–d1,b2–d2). For Fluid E, the 35 wt% PESA made the surface of the filter cake dense and extremely smooth. In addition, the long cracks in the filter cake were caused by drying and were not present in the wet filter cake (Figure 6e1,e2). As shown in Figure 6a3–e3, EDS analysis revealed that with increasing PESA concentration, both the Na and O contents in the filter cakes exhibited increasing trends, while the Ba content in the filter cakes exhibited the opposite trend, indicating a decrease in the barite content and an increase in the PESA content in the filter cakes. The results of this analysis provided a basis for elucidating the adsorption mechanism of a water-soluble polymer with a low molecular weight on particle surfaces and filter cakes, demonstrating that increasing PESA concentration made the filter cake denser.

**Figure 7:**
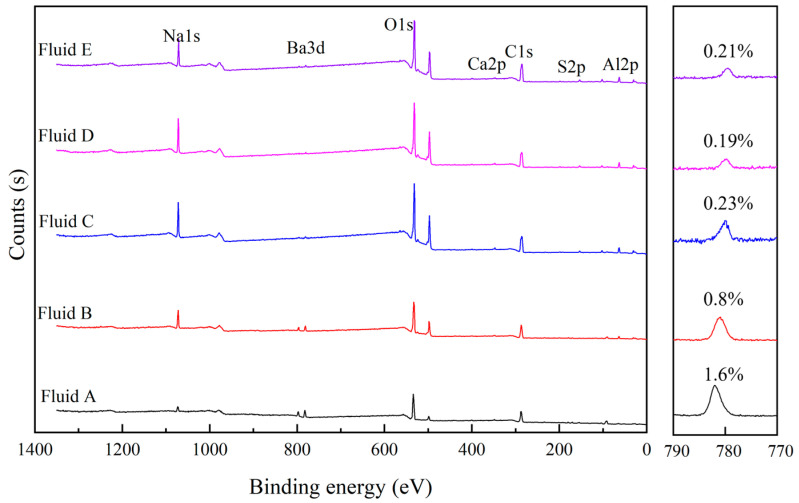
XPS survey spectra of the filter cake.

To analyze the mechanism of action of PESA in filter cakes, XPS was carried out to investigate the elemental compositions of the filter cakes. The XPS survey spectra of the filter cakes are shown in Figure 7. According to the peak areas of the elements and atomic concentrations, with increasing PESA concentration, the contents of both Na and O in the filter cakes increased, while the content of Ba in the filter cakes showed the opposite trend. The elements O and Na in the PESA molecules accounted for a large proportion, and the main component of barite was BaSO_4_. Therefore, the changes in the Na, O, and Ba contents indicated that the barite content decreased and the PESA content increased in the filter cakes with increasing PESA concentration, making the filter cake denser, which was consistent with the general trends in the SEM and EDS results. The contents of C, O, and Ba in the filter cakes determined via XPS analysis and EDS analysis were different, but the general trends were the same. In particular, the Ba content obtained from the XPS analysis was lower than that obtained from the EDS analysis. These differences can be attributed to the fact that the surface of the filter cake was covered with the polymer (PESA) and that the XPS detection depth was 10 nm [59]; thus, there was a large difference in the results obtained from the XPS analysis and EDS analysis, which further demonstrated the role of PESA in dense filter cake formation and filtration control. The barite particles on the surface of the filter cakes were mainly covered by PESA molecules, and the filter cakes became denser and smoother as the PESA concentration in the WBDFs increased. In addition, PESA bound free water, preventing the liquid from penetrating the filter cake. With increasing PESA concentration, more polymers adsorbed on and encapsulated the solid particles, making the filter cake denser, which also explained the smooth morphology of the filter cake from another perspective.

### 3.6. Discussion Mechanism of Lubricity and Filtration Control

**Figure 8:**
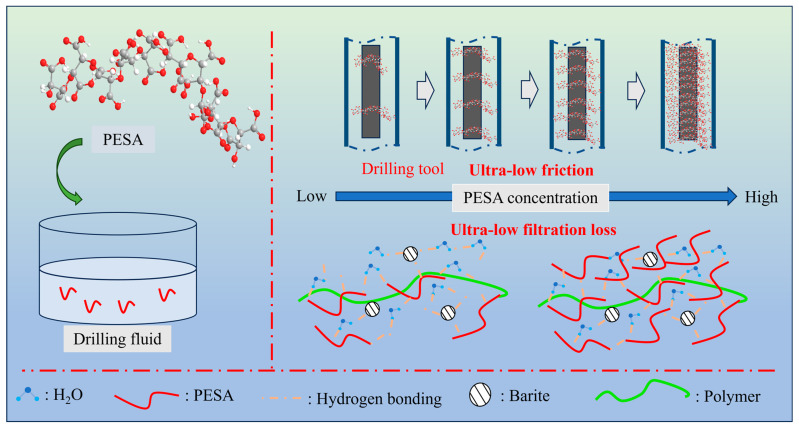
Schematic diagram of the PESA mechanism in WBDFs.

The probable mechanism that roles of PESA was proposed in Figure 8. In fact, our experiment further confirmed that an aqueous solution with a high concentration of PESA has an ultra-low COF. The first reasonable explanation for this result was that PESA adsorption occurs on the steel–steel friction surfaces. The second reasonable explanation for the result was that the solid particles adsorbed by PESA reduced friction, which further reduced the COF. The higher the PESA concentration was, the more obvious the adsorption on the surface of the solid particles and steel, which was more conducive to a reduction in the COF. The microscopic XPS and EDS analyses also verified this phenomenon. The quality of the filter cake formed by WBDFs under a pressure difference determines the filtration loss [60]. As the main filtration reducer, starch was mainly adsorbed with other additives to form the skeleton structure of the filter cake [61], and barite mainly exists in the form of a physical filler in the filter cake. There was no bentonite to provide the skeleton structure in Fluid A, resulting in large pores and a large filtration loss. From Fluid B to Fluid E, the amount of PESA ranged from 5 to 15, 25, and 35 wt%, and the pores in the filter cakes gradually disappeared with increasing PESA concentration, which was mainly due to the interaction between PESA and particles such as starch and barite. On the one hand, the molecular chains of PESA can fill the pores of the filter cake; on the other hand, PESA adsorbs on the surface of barite particles and then interacts with starch particles to strengthen the adsorption between the particles. EDS analysis revealed that the content of barite in the filter cake decreased with increasing PESA concentration, indicating that the barite particles in the filter cake were replaced by PESA molecules (Figure 6a3–e3). Furthermore, the content of free water in the drilling fluid decreased with increasing PESA concentration, and PESA molecules could spontaneously bind free water [62]. Under the effect of a pressure difference, the resistance of PESA molecules with high viscosity and the bound free water through the dense filter cake could be greater, which was manifested by a decrease in the filtration loss.

### 3.7. Environmental Sustainability and Economic Viability

PESA is recognized as an environmentally sustainable polymer that is widely used in industries such as water treatment and mineral flotation [28]. It is foreseeable that SP-WBDFs, which are formulated by combining PESA with renewable natural polymers like xanthan gum, starch, and cellulose, will also be characterized by excellent environmental sustainability [47]. In certain regions, the disposal of drilling cuttings generated by OBDFs and SBDFs has severely constrained oil and gas exploration and production. This is primarily due to the potential environmental pollution risks posed by OBDFs and SBDFs. In contrast, SP-WBDFs formed with PESA will not face these ecological risks [63]. A superior option is provided for the extraction of oil and gas resources in more eco-friendly manner. The cost per cubic meter of SP-WBDFs based on PESA is expected to fall between OBDFs, SBDFs, and traditional WBDFs. Although the cost is slightly higher compared to traditional WBDF, drilling and environmental issues that cannot be resolved by traditional WBDF can be addressed by SP-WBDFs, and OBDFs and SBDFs can even be replaced. In other words, SP-WBDFs are considered economically viable.

## 4. Conclusions

In summary, the utilization of high-concentration materials to achieve super-performance in WBDFs was developed, which was well illustrated by the concentration and adsorption of water-soluble polymers. The experimental results confirmed that the ability to inhibit the hydration and dispersion of bentonite gradually increased with increasing PESA concentration in the aqueous solutions. Under the premise of good rheology, the WBDFs with a high concentration of PESA as a continuous phase demonstrated an ultra-low friction coefficient and effective filtration loss control, as mutually proven by the macro performance and microanalysis. The following conclusions were drawn:

1. The COF of the aqueous solution with 25 wt% PESA was close to that of the mineral oi, while the COF of the aqueous solution with 35 wt% PESA was 0.013, significantly lower than the 0.037 observed for mineral oil.
2. Water contaminated with over 300 g/L of bentonite experienced significant thickening, making it impossible to measure AV of the resulting mixture. In contrast, the aqueous solutions with over 15 wt% PESA exhibited excellent performance in inhibiting bentonite hydration and dispersion. The AV of the aqueous solutions with over 25 wt% PESA can still be measured, even after contamination with 600 g/L of bentonite.
3. The SP-WBDF with 35 wt% PESA exhibited excellent rheological properties that meet drilling requirements, achieving ultra-low friction and minimal filtration loss; the COF, API filtration loss, and HTHP filtration loss were 0.016, 0.4 mL, and 3.0 mL, respectively.

## Figures and Tables

**Table 1:** Formulation of the WBDFs.

Fluid Compositions		Unit	Fluid A	Fluid B	Fluid C	Fluid D	Fluid E	Function
Base fluid *	Fresh water	g	100	98.3	96.2	88.0	84.5	Continuous phase
	PESA **	g	0	5.2	17.0	29.4	45.5	
NaOH		g	0.1	0.1	0.1	0.1	0.1	Alkalinity regulator
PAC-LV		g	0.5	0.5	0.5	0.5	0.5	Filtration reducer
PHPA		g	0.1	0.1	0.1	0.1	0.1	Shale inhibitor
Xanthan gum		g	0.3	0.3	0.3	0.3	0.3	Flow pattern regulator
Starch		g	2.0	2.0	2.0	2.0	2.0	Filtration reducer
Barite		g	25.0	25.0	25.0	25.0	25.0	Weighting agent

*: The volume of the base fluid is 100 mL. **: The corresponding concentrations of PESA in the base fluids are 0%, 5 wt%, 15 wt%, 25 wt%, and 35 wt%, respectively.

## Data Availability

The original contributions presented in this study are included in the article. Further inquiries can be directed to the corresponding author.
